# Achilles tendon morphology adaptations in chronic post-stroke hemiparesis: a comparative analysis with neurologically intact controls

**DOI:** 10.3389/fspor.2024.1498333

**Published:** 2025-01-07

**Authors:** Jing Nong Liang, Greg Bashford, Kornelia Kulig, Kai-Yu Ho

**Affiliations:** ^1^ Department of Physical Therapy, University of Nevada, Las Vegas, Las Vegas, NV, United States; ^2^ Department of Biological Systems Engineering, University of Nebraska-Lincoln, Lincoln, NE, United States; ^3^ Division of Biokinesiology & Physical Therapy, University of Southern California, Los Angeles, CA, United States

**Keywords:** Achilles tendon, post-stroke hemiparesis, walking speed, muscle tone, morphology

## Abstract

**Introduction:**

In individuals with chronic post-stroke hemiparesis, slow walking speed is a significant concern related to inadequate propulsion of the paretic limb. However, an overlooked factor is this population's altered morphology of the Achilles tendon, which may compromise the propulsive forces by the paretic limb. This study aimed to explore changes in Achilles tendon morphology, including gross thickness and intra-tendinous collagen fiber bundle organization, following stroke-induced brain lesions.

**Methods:**

Fifteen individuals with chronic post-stroke hemiparesis (at least 6 months post-stroke) and 19 neurologically intact controls participated. Ultrasound imaging was used to evaluate Achilles tendon thickness and collagen organization in the paretic and non-paretic limbs of post-stroke participants, as well as in the right limb (control limb) of the neurologically intact control group.

**Results and discussion:**

Compared to control individuals, the paretic limb in individuals post-stroke showed increased tendon thickness at the Achilles tendon insertion and 2 cm above it. The collagen fiber bundle at the Achilles tendon insertion of the paretic limb showed reduced organization compared to that in the control limb. Individuals post-stroke also exhibited slower walking speed, and increased plantarflexor muscle tone in the paretic limb compared to controls. In conclusion, individuals with chronic post-stroke hemiparesis demonstrated tendon thickening and collagen disorganization in the paretic limb, particularly at the insertion site of the Achilles tendon, likely due to an abnormal loading environment influenced by increased plantarflexor muscle tone, muscle co-activation, and muscle disuse and atrophy. These changes may increase tendon compliance, impair force transmission and propulsion, and contribute to slower walking speed. Addressing Achilles tendon integrity should be incorporated as a component of strategies to improve neuromuscular control in this population.

## 1 Introduction

Stroke is a leading cause of long-term disability, significantly impacting mobility and quality of life for millions of survivors. Restoring walking ability is a primary goal of post-stroke rehabilitation, as it is crucial for independence and participation in daily activities (1–3). However, despite regaining the ability to walk, many individuals continue to experience gait impairments, especially slow walking speed, even years after stroke (4, 5). This persistent slow walking speed is a major concern because it limits community reintegration and increases the risk of secondary health complications, falls, and dependence on caregivers (1, 6, 7). A critical factor contributing to slow walking speed after stroke is insufficient propulsion generated by the paretic leg (8, 9). Propulsion, the force that drives the body forward, is primarily produced by the ankle plantarflexor muscles (gastrocnemius and soleus) during the push-off phase of gait. Weakness in these muscles, altered muscle activation patterns, and increased muscle tone, are key factors underlying insufficient propulsion (10, 11). To execute effective walking, locomotor control requires the integration and coordination of descending central drive, spinal circuits, and the force transmission capabilities of muscle-tendon units. Studies suggest that the Achilles tendon may undergo significant adaptations following stroke (12–14), which may impact its ability to effectively transmit forces, thus negatively affecting propulsion and step-to-step transitions during gait.

In individuals post-stroke, the Achilles tendon of the paretic limb is exposed to a complex loading environment. Increased plantarflexor muscle tone (12, 14) and disrupted timing of agonist and antagonist muscle activity, along with inappropriate co-activation of lower limb muscles (15), contribute to sustained low-grade forces within the Achilles tendon. Despite this, force transmission from the plantarflexor muscles is compromised due to muscle atrophy and disuse in persons post-stroke (13). These abnormal forces may influence tendon morphology and mechanical properties, as tendons are highly responsive and adaptive to mechanical loading (12, 13, 16). However, existing literature presents inconsistent findings on Achilles tendon morphology, particularly concerning tendon length, size [e.g., cross-sectional area (CSA) and thickness], and mechanical properties in individuals post-stroke. Regarding tendon length, some studies reported increased tendon length in the paretic limb, potentially due to muscle contracture and higher muscle fascicle stiffness, which may cause dislocation of the muscle-tendon junction. For instance, Zhao et al. found that the paretic limb's Achilles tendon was 6% longer than the non-paretic limb (12). They suggested that as the calf muscles shorten and become stiffer post-stroke, the muscle-tendon junction shifts proximally, resulting in a lengthened tendon. In their next study, Zhao and colleagues corroborated this finding, reporting a 5% increase in tendon length in the paretic limb (13). They suggested that muscle atrophy, impaired neural control, and reduced movement post-stroke might contribute to shorter and stiffer muscle fascicles, which, in turn, exert a prolonged stretch on the tendon, leading to tendon elongation. However, other studies found no significant difference in tendon length between the paretic and non-paretic limbs. For example, Freire et al. compared Achilles tendon length in stroke survivors’ paretic and non-paretic limbs with those of neurologically intact controls and found no significant differences between groups (14). Similarly, Dias et al. reported no difference in tendon length between stroke survivors’ paretic and non-paretic limbs (16). These differences may stem from variations in age, activity level and/or the time since the stroke incidence among participants of these studies.

Regarding tendon size, the available literature also presents conflicting evidence. Some studies observed a reduced Achilles tendon CSA in the paretic limb compared to the non-paretic limb and neurologically intact controls. Dias et al. (16) reported an 18% decrease in Achilles tendon CSA in the paretic limb of individuals post-stroke compared to controls, while Zhao et al. (13) observed a 5% reduction in CSA in the paretic limb compared to the non-paretic limb in individuals post-stroke. The authors posit that muscle atrophy and reduced force transmission from muscle contractions to the tendon, possibly due to spasticity, might contribute to the reduced CSA. However, Zhao et al. (12) did not find a statistically significant difference in CSA between the paretic and non-paretic limbs. In our recent preliminary study (17), we observed thicker Achilles tendon at the insertion site in the paretic limbs of stroke survivors compared to control limbs of neurologically intact controls.

Several studies have investigated the mechanical properties of the Achilles tendon in the paretic limb of individuals after stroke with conflicting results. While Freire et al. (14) found no differences in tendon stiffness between paretic, non-paretic and control limbs, most studies reported decreased stiffness and Young's modulus in the paretic limb compared to both the non-paretic limb in individuals post-stroke (12, 13, 16) and the control limb of neurologically intact individuals (16). The decreased stiffness may be attributed to a reduction in collagen content, likely resulting from diminished force transmission to the tendon due to reduced muscle contraction (16). Limitations of previous studies on tendon morphology and mechanical properties include insufficient examination of intra-tendinous structure (e.g., collagen organization) and tendon thickness (13, 16), small sample sizes (17), and/or a lack of comparisons between paretic and non-paretic limbs in individuals post-stroke and those of neurologically intact controls (12, 13).

Ultrasound imaging commonly assesses gross morphological changes in diseased tendons, such as increased thickness (18–21) and CSA (22). It can also assess intra-tendinous morphology (21, 23). Bashford et al. (24) introduced spatial frequency analysis (SFA) to evaluate tendon collagen structure, represented by the “speckle pattern” on ultrasound images (20, 25). SFA can infer the structure of a group of collagen fiber bundles through frequency analysis of the speckle pattern. Briefly, the presence of higher spatial frequency content in the speckle pattern is indicative of increased organization. SFA operates in two dimensions, making it less dependent on probe angle than axial-only spectrum analysis. Among SFA parameters, peak spatial frequency radius (PSFR) is the most reported; lower PSFR indicates collagen disarray, while higher values suggest better collagen organization (24, 25). Kulig et al. (25) found healthy tendons have a mean PSFR of 2.07 mm^−1^, while degenerated tendons average 1.55 mm^−1^. For these reasons, a larger-scale study is needed to examine both the intra-tendinous and gross morphology of the Achilles tendon, comparing the paretic and non-paretic limbs of individuals with stroke to those of neurologically intact controls.

A systematic review by van der Vlist et al. (26) identified nine clinical risk factors for Achilles tendinopathy, indicating that decreased plantarflexor strength and abnormal gait patterns with reduced forward propulsion are two key biomechanical contributors to the development of this condition, which are also particularly prevalent in individuals post-stroke (8). Moreover, it is important to consider that individuals post-stroke may have an increased vulnerability to insertional Achilles tendinopathy (27) due to theoretical associations with metabolic syndrome—factors hypothesized to contribute to the condition's onset (28–30). Our preliminary study involving two individuals chronically post-stroke and two neurologically intact controls revealed potential associations among increased plantarflexor muscle tone, Achilles tendon thickening, and reduced propulsive forces during gait in the paretic limb of individuals post-stroke (17). An abnormal loading environment on the Achilles tendon is thought to alter its morphology, leading to increased tendon compliance, and subsequently causing delayed and reduced force transmission during gait. These changes may lead to poorly timed and insufficient propulsion (12, 17, 31). By investigating the morphological changes of the Achilles tendon in individuals post-stroke in the chronic phase of recovery, this study aimed to contribute to a more comprehensive understanding of the factors related to gait impairments after stroke. This knowledge is essential for developing effective rehabilitation strategies to improve walking speed, mobility, and quality of life for stroke survivors.

Therefore, the primary objective of this cross-sectional study was to examine differences in the gross morphology of the Achilles tendon, explicitly focusing on thickness, as well as intra-tendinous morphology related to collagen fiber bundle organization, using ultrasound imaging between individuals with chronic post-stroke hemiparesis and neurologically intact controls. We hypothesized that compared to neurologically intact controls, individuals with chronic post-stroke hemiparesis would show alterations in Achilles tendon morphology in the paretic limb. Additionally, the secondary aim was to evaluate plantarflexor muscle tone, lower extremity function, and walking speed in stroke participants to enhance the clinical relevance of our findings. We anticipated that individuals post-stroke would exhibit increased plantarflexor muscle tone, reduced lower extremity function, and decreased walking speed.

## 2 Methods

### 2.1 Participants

Before the study, we conducted a sample size calculation, projecting an effect size (Cohen's d) of 1.0 for differences in intra-tendinous morphology (collagen fiber bundle organization) between groups with and without tendon pathology, based on findings from previous research (20). Using this estimated effect size, an alpha level of 0.05, and a power of 0.80, our analysis determined that that 28 participants (14 per group) would be required. For tendon thickness differences between stroke and control groups, we projected an effect size of 2.1 based on our previous work (17). The analysis for tendon thickness indicated that 10 participants (5 per group) would be sufficient, using the same alpha level and power. Ultimately, we selected a sample size of 28 participants, deemed adequate to detect differences in Achilles tendon morphology, specifically regarding both thickness and collagen fiber bundle organization.

Inclusion criteria for individuals with chronic post-stroke hemiparesis comprised the following: age over 18 years old, a minimum of six months after a single, unilateral, cortical, or subcortical stroke with residual hemiparesis, the ability to walk 10 meters overground independently without assistive devices, and the ability to follow cues and adhere to instructions. Inclusion criteria for neurologically intact controls were age over 18 years old, able to complete the 10-Meter Walk Test, and ability to follow cues and adhere to instructions.

Individuals post-stroke were excluded if they had a history of cerebellar stroke(s), lower extremity surgery, Achilles tendon tendinopathy (past or present), pregnancy or suspected pregnancy, had other neurological diagnoses, or had used medications to manage spasticity or received Botox injection in the lower extremity within the past six months. Control participants were excluded if they were pregnant or suspecting pregnancy, had a history of lower extremity surgery, had a history and/or current Achilles tendon tendinopathy, or had a diagnosis of any neurological disorder. Control participants were matched for sex and age, with age differences kept within 10% compared to their counterparts in the stroke group.

Participant recruitment and data collection took place between 2019 and 2022 in the Las Vegas area. Before their involvement in the study, we obtained signed informed consent from each participant. The protocol was approved by the Institutional Review Board at the University of Nevada, Las Vegas (Protocol #: 1250168).

### 2.2 Procedures

#### 2.2.1 Clinical outcome measurements

Prior to tendon morphology measurements, we assessed overground walking speed using the 10-Meter Walk Test (32), lower extremity motor function using Fugl-Meyer assessment of the lower extremity (FMA-LE) (33), and plantarflexor muscle tone using Modified Ashworth Scale (MAS) (34).

Trained investigators assessed the FMA-LE for each participant within the stroke group, with 34 being the maximum possible score for lower extremity motor function (33). The 10-Meter Walk Test was administered to both the stroke-impaired and neurologically intact control groups, involving two trials of overground walking trials at self-selected speed for each participant. The average of two trials was documented to show ambulatory capacity at self-selected walking speed (32). To assess plantarflexor muscle tone, individuals post-stroke underwent MAS assessments involving passive dorsiflexion of the ankle, where zero indicated normal muscle tone, and higher scores represented elevated spasticity and increased resistance to passive movement. A score of 4 on the MAS indicated rigidity during the assessment (34).

#### 2.2.2 Tendon morphology measurements

Bilateral Achilles tendon morphology, including thickness and collagen fiber bundle organization, was assessed using ultrasound imaging (GE LOGIQ-e, GE Healthcare, Milwaukee, WI, USA). Participants were positioned prone on a treatment table with the knee extended and the ankle positioned in neutral (0 degree between shank and foot segments). Using a preset for ankle musculoskeletal examination at a depth of 2 cm, we employed a linear array ultrasound transducer (GE 12L-RS, bandwidth 5–13 MHz, width 38.4 mm, GE Healthcare, Milwaukee, WI, USA) to capture longitudinal images at three specific locations: the Achilles tendon insertion on the calcaneus, 2 cm above the insertion, and 4 cm above the insertion (23).

### 2.3 Image analysis

**Figure 1:**
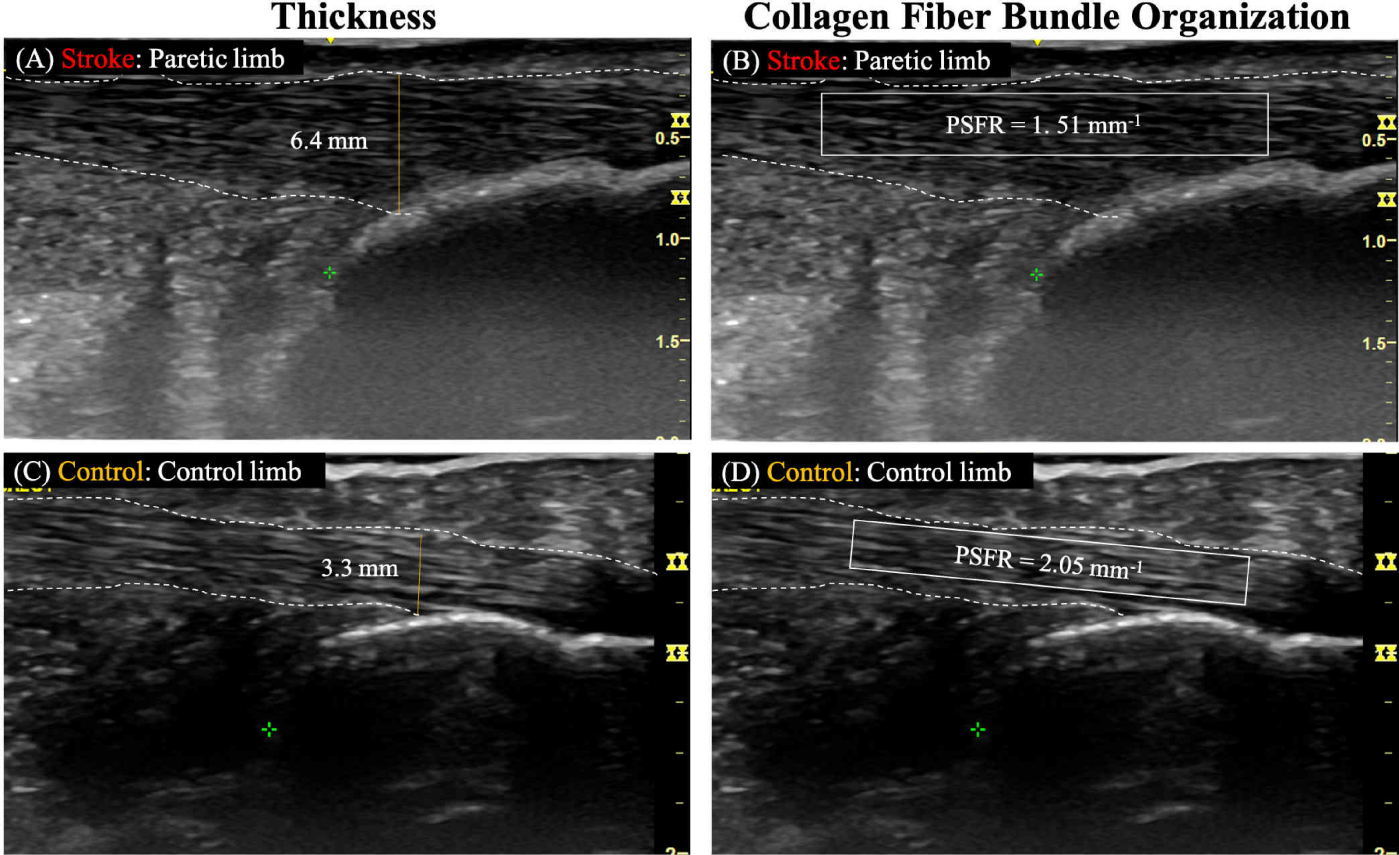
Representative ultrasound images analyzing achilles tendon thickness and collagen fiber bundle organization. Panels **(A,B)** show the paretic limb of a participant with chronic post-stroke hemiparesis, and panels **(C,D)** illustrate the control limb of a neurologically intact control participant. White dashed lines outline the tendon borders and the yellow solid line represents the tendon thickness. The solid-lined box indicates the region of interest (ROI) for spatial frequency analysis (SFA).

The thickness of the Achilles tendon at 2 cm and 4 cm above the insertion were determined as the perpendicular distance between the borders outlining the Achilles tendon at the longitudinal image's center (17, 23). For the thickness at Achilles tendon insertion, the measurement was taken as the perpendicular distance between the borders at the Achilles tendon-calcaneus intersection (Figures 1A,C) (17).

To quantify collagen fiber bundle organization (PSFR) on each longitudinal image (Figures 1B,D), SFA was performed using a custom MATLAB program, following the methodology described in previous studies (20, 21, 23). In this process, a region of interest (ROI) was selected to encompass the maximum visible tendon and peritenon area while excluding the distal and proximal image boundaries. This selection aimed to minimize potential errors arising from the curvature of the distal and proximal tendon. For each ROI, SFA was performed on all possible 32 × 32 pixel (∼2.0 mm) sub-images (‘kernels’). Briefly, 2D Fast Fourier Transforms (FFTs) were applied to each kernel after zero-padding to 128 × 128 samples to increase frequency sampling. A 2D high pass filter (−3 dB cut-off about 1.0 mm^−1^) was applied to attenuate low spatial frequency artifacts. Spatial frequency parameters were extracted and averaged over all kernels of the ROI, thereby analyzing spatial frequencies in both the axial and lateral directions.

#### 2.3.1 Image acquisition and analysis reliability

Before the study commenced, senior sonographer K.H. conducted comprehensive training for the investigators, focusing on image acquisition and analysis techniques for Achilles tendon thickness and PSFR. The investigators were assigned specific tasks only after demonstrating repeatability in image acquisition and analysis through a reliability study.

To assess intra-rater reliability in image acquisition, researchers A.C. and M.S. conducted two data collection sessions with 5 participants, each session separated by a one-week interval. For image analysis reliability, undertaken by investigators A.C., M.S., and T.D., measurements of images from five individuals were taken on two separate days, spaced a week apart. Intra-class correlation coefficient (ICC) and standard error of measurement (SEM) were utilized to measure intra-rater reliability for image acquisition and analysis, with ICC values categorized as poor (<0.4), fair (0.4–0.7), good (0.7–0.9), and excellent (>0.9) (35). The SEM was calculated by multiplying the standard deviation by the square root of 1 minus the reliability coefficient (23).

The investigators responsible for image acquisition demonstrated good to excellent intra-rater reliability in measuring tendon thickness (ICC = 0.852–0.997; SEM = 0.039–0.092 mm) and PSFR (ICC = 0.731–0.828; SEM = 0.056–0.062 mm^−1^). Similarly, those involved in image analysis exhibited good to excellent intra-rater reliability for measuring tendon thickness (A.C. and M.S.; ICC = 0.808–0.999; SEM = 0.011–0.105 mm) and PSFR (A.C. and T.D.; ICC = 0.993–0.977; SEM = 0.024–0.031 mm^−1^).

### 2.4 Statistical analysis

The outcome measures of this study included thickness and PSFR of the Achilles tendon. The paretic and non-paretic limbs were included for participants with post-stroke hemiparesis. The right limb was used for neurologically intact participants to represent the control limb. We conducted a Shapiro-Wilk test and the results indicated that the outcome variable data followed a normal distribution. Therefore, one-way ANOVAs and post-hoc pairwise comparisons with Bonferroni corrections were used to compare thickness and PSFR of the Achilles tendon among the three limbs (paretic, non-paretic, and control limbs). All statistical analyses were performed using SPSS software (ver. 27, International Business Machines Corp. New York, USA). A significant difference was defined as *p* ≤ 0.05.

## 3 Results

### 3.1 Participant characteristics

Table 1 shows the characteristics of participants with chronic post-stroke hemiparesis and neurologically intact controls. Both groups had similar sex distribution (47% females and 53% males): the chronic post-stroke hemiparesis group comprised 7 females, while the control group had 9 females. Both groups had similar age, height, and weight. Participants with chronic post-stroke hemiparesis had a slower overground self-selected walking speed compared to control participants. On average, participants with chronic post-stroke hemiparesis had a MAS score of 0.8, suggesting slight increase in ankle plantarflexor muscle tone.

**Table 1:** Participant characteristics.

	Participants with chronic post-stroke hemiparesis (*n* = 15)	Neurologically intact controls (*n* = 19)	*p*
Sex	7 females; 8 males	9 females; 10 males	NA
Age, y	59.29 (10.38), 53.70–64.80	55.32 (14.52), 47.40–62.10	0.31
Height, cm	168.70 (6.00), 153.70–183.60	169.60 (8.90), 163.60–175.60	0.84
Weight, kg	79.30 (13.20), 46.50–112.10	71.30 (15.00), 61.20–81.40	0.42
Years post-stroke	6.70 (5.40), 3.80–9.70	NA	NA
Self-selected overground walking speed, m/s	0.88 (0.18), 0.45–1.32	1.44 (0.47), 1.29–1.71	0.01*
Fugl-Meyer assessment of the lower extremity (FMA-LE)	22.57 (5.30), 18.70–25.10	NA	NA
Modified Ashworth Scale (MAS)	0.80 (0.45), 0.20–1.20	NA	NA

The data were displayed as mean (standard deviation), and 95% confidence interval.

### 3.2 Tendon morphology

#### 3.2.1 Thickness

ANOVA results revealed a statistically significant difference in tendon thickness both 2 cm above the insertion [F(2, 46) = 3.64, *p* = 0.03] and at the insertion of the Achilles tendon [F(2, 46) = 5.68, *p* < 0.01]. *Post hoc* analyses, when compared to the control limb showed that the paretic limb exhibited significantly greater tendon thickness at both 2 cm above insertion (*p* = 0.03) and at the insertion (*p* < 0.01). No statistically significant difference was observed in tendon thickness between the non-paretic and paretic limbs among individuals with post-stroke hemiparesis at either 2 cm above the insertion (*p* = 1.00) or at the insertion of the Achilles tendon (*p* = 0.11). Moreover, no statistically significant difference in tendon thickness was found among the three limbs at 4 cm above the insertion of the Achilles tendon [F(2, 46) = 1.38, *p* = 0.26] (Table 2).

**Table 2:** Comparisons of tendon thickness and peak spatial frequency radius (PSFR) at the achilles tendon insertion, 2 cm above the insertion, and 4 cm above the insertion between the paretic and non-paretic limbs of participants with post-stroke hemiparesis and the control limb of neurologically intact control participants.

	Control limb	Non-paretic limb	Paretic limb	Effect size	*p*
Thickness (mm)					
4 cm above insertion	4.58 (0.07), 4.25–4.92	5.00 (0.13), 4.29–5.70	5.10 (0.07), 4.66–5.46	0.06, 0.00–0.19	0.26
2 cm above insertion	4.37 (0.06), 4.09–4.64	4.73 (0.07), 4.37–5.09	4.93 (0.07), 4.57–5.30^a^	0.14, 0.00–0.30	0.03*
At insertion	4.08 (0.07), 3.76–4.41	4.36 (0.07), 3.96–4.75	4.92 (0.08), 4.47–5.38^a^	0.20, 0.02–0.37	<.01*
PSFR (mm^−1^)					
4 cm above insertion	1.95 (0.24), 1.83–2.06	1.81 (0.19), 1.70–1.92	1.89 (0.20), 1.78–2.00	0.07, 0.00–0.21	0.19
2 cm above insertion	1.91 (0.27), 1.78–2.04	1.87 (0.37), 1.67–2.07	1.85 (0.20), 1.74–1.96	0.01, 0.00–0.08	0.82
At insertion	1.94 (0.32), 1.78–2.09	1.78 (0.26), 1.64–1.93	1.70 (0.21), 1.59–1.82^a^	0.13, 0.00–0.29	0.04*

The data of thickness and PSFR were displayed as mean (standard deviation), and 95% confidence interval. Effect sizes, along with their 95% confidence intervals, are also provided.

^a^ Denotes a significant difference from the control limb.

* Denotes a significant difference using one-way ANOVA with repeated measures.

#### 3.2.2 Collagen fiber bundle organization (peak spatial frequency radius)

ANOVA results revealed a statistically significant difference in the PSFR at the insertion of the Achilles tendon [F(2, 46) = 3.32, *p* = 0.04]. Subsequent *post hoc* analyses showed that the paretic limb exhibited a lower PSFR at the insertion of the Achilles tendon compared to the control limb (*p* = 0.04). There was no statistically significant difference in PSFR at the insertion of the Achilles tendon between the non-paretic and paretic limbs in individuals with post-stroke hemiparesis (*p* = 1.00). Furthermore, no statistically significant difference in PSFR was identified among the three limbs at both 2 cm [F(2, 46) = 0.20, *p* = 0.82] and 4 cm above the insertion of the Achilles tendon [F(2, 46) = 1.75, *p* = 0.19] (Table 2).

## 4 Discussion

This study sought to investigate the alterations in Achilles tendon morphology, with a specific focus on thickness and collagen organization, following stroke-induced brain lesions in the chronic phase of recovery. As hypothesized, individuals with chronic post-stroke hemiparesis exhibited changes in tendon morphology, including increased thickness both 2 cm above the insertion and at the insertion site of the Achilles tendon in comparison to the control limb of neurologically intact individuals. Moreover, individuals with chronic post-stroke hemiparesis demonstrated a decreased PSFR at the insertion of the Achilles tendon, suggesting lesser collagen fiber bundle organization, when compared to the control limb of neurologically intact individuals. Additionally, individuals post-stroke walked slower than neurologically intact controls. They also exhibited increased plantarflexor muscle tone and diminished lower extremity function, which aligned with our expectations.

A key observation in this study is that the primary area of tendon changes was concentrated at the insertion point of the Achilles tendon in the paretic limb of individuals with post-stroke hemiparesis. This agrees with our preliminary case study (17), where we observed increased thickness at the calcaneal insertion of the paretic Achilles tendon, compared to similar thickness between limbs in neurologically intact controls. Dias et al. examined tendon length and CSA, reporting that the paretic limb showed reduced CSA compared to the neurologically intact control limb and the non-paretic limb, which also had reduced CSA compared to the neurologically intact control limb (16). Although the exact location of these CSA measurements was not specified, the study's focus on overall mechanical properties suggested these values likely represent averages across the tendon rather than measurements at specific regions (16). Zhao et al. focused on tendon length, CSA, stiffness, Young's modulus, and hysteresis (12, 13), but like Dias et al., they primarily reported mean differences between limbs without specific regional data. Zhao et al. did, however, note that the soleus muscle-tendon junction might shift proximally in the paretic limb (12). In summary, although existing literature offers valuable insights into alterations of the Achilles tendon post-stroke, it lacks a consistent focus on specific regions, particularly the insertion point. This study is the first to examine and report findings across three distinct regional locations within the Achilles tendon in the post-stroke population. Prior studies predominantly report mean values from multiple tendon sites, limiting the ability to make direct comparisons with our findings. These observations underscore the need for future research to investigate regional variations in tendon morphology after stroke.

The time after stroke, including factors such as chronicity and variability, and spasticity levels are potentially critical factors influencing Achilles tendon properties in individuals post-stroke. Our study, with participants averaging 6.4 years post-stroke and presenting with mild plantarflexor hypertonia (MAS score of 0.8), offers a distinct perspective compared to the available research (Table 1). Dias et al. (16) included individuals who were at least one year post-stroke, but did not specify an average duration. Zhao et al. (12) included participants with a mean of 12 years after stroke, while their later work (13) examined individuals with a mean of 9.5 years after stroke (range 1.8–20.8 years). These studies, along with ours, represent a spectrum of time after stroke, highlighting the importance of considering this factor when comparing findings. The observed changes in tendon properties are also likely influenced by levels of spasticity, which can vary significantly across studies. Zhao et al. (12) reported a mean MAS score of 1.6, indicating moderate spasticity, while their later study (13) noted an even higher mean MAS score of 2.5. Dias et al. (16) included participants with an average MAS score of 1.5, similar to Zhao et al. (12). In contrast, our participants exhibited lower plantarflexor muscle tone, with an average MAS score of 0.8. This difference in spasticity levels may contribute to the observed discrepancies in findings. The observations from these studies and our work highlight the complex interplay between factors such as stroke chronicity and muscle hypertonia, with tendon morphology adaptations. Longitudinal analyses are essential to capture tendon morphology changes at multiple sites over time. Understanding these relationships is crucial for developing targeted rehabilitation interventions aimed at optimizing tendon function and improving functional outcomes in individuals post-stroke. Further research utilizing longitudinal designs and controlled spasticity levels is necessary to fully elucidate these dynamics.

While we did not directly assess the mechanical properties of the Achilles tendon in our study, the increased tendon compliance frequently reported in individuals post-stroke (12, 13, 16) may be linked to the tendon morphological changes observed in our findings. In addition, we observed reduced PSFR in the paretic Achilles tendon of individuals post-stroke. Previous research has shown that lower PSFR is associated with decreased tendon stiffness and Young's modulus in degenerated Achilles tendons (25). This adaption at the Achilles tendon insertion in the paretic limb may stem from collagen fiber disorganization and reduced collagen content (16), which result from a complex, abnormal loading environment characterized by factors such as increased muscle tone (12, 14), muscle co-activation (15), and muscle atrophy and disuse (13). It is possible that the morphological changes in the Achilles tendon among individuals with post-stroke hemiparesis may impair the propulsive force generated by the muscle-tendon unit of the paretic ankle plantarflexors. This deficiency, attributed to excessive tendon compliance, likely contributes to reduced walking speed in this cohort. Additionally, it is important to note that decreased plantarflexor strength and abnormal gait pattern with diminished forward propulsion during gait are recognized risk factors for Achilles tendinopathy (26). This insufficient propulsion may further compromise tendon morphology and health in individuals post-stroke.

Furthermore, the alterations observed at the tendon insertion in individuals post-stroke may be associated with metabolic syndrome. Metabolic syndrome is a cluster of conditions that includes central obesity, hypertension, hyperglycemia and dyslipidemia, contributes to systemic inflammation and altered tissue metabolism (30, 36). This multifaceted condition has been identified as a major contributor to cardiovascular diseases, including stroke (30, 37). Metabolic syndrome affects 62% of ischemic stroke patients (38). While each component of metabolic syndrome is associated with an increased risk of stroke, research suggests that truncal obesity and hypertension are independent risk factors (37, 38). Metabolic syndrome also has significant implications for tendon health. The chronic inflammation associated with obesity can impair tendon healing and increase the risk of tendinopathy. Specifically, obesity elevates the risk of Achilles tendinopathy, likely due to the influence of adipokines and the systematic inflammatory response triggered by adipose tissue (30). The Achilles tendon may be particularly susceptible to the effects of metabolic syndrome due to its role in weight-bearing activities and the high mechanical demands placed upon it during locomotor activities, such as walking and running (39–42). Anatomically, the Achilles tendon has a relatively higher blood supply at its insertion point (43, 44), making this area more reliant on adequate circulation. In conditions associated with metabolic syndrome, reduced blood circulation to the Achilles tendon, can compromise the tendon's ability to recover from mechanical fatigue and increase susceptibility to injury (30). Consequently, the Achilles tendon, particularly at its insertion site, is likely at a heightened risk for ischemia and impaired healing in the presence of metabolic dysfunction. In line with existing literature, the recorded PSFR values for both the paretic and non-paretic limbs within the stroke group, measuring 1.70 mm^−1^ and 1.89 mm^−1^ (Table 2), were notably lower compared to the healthy Achilles tendon (2.07 mm^−1^) documented in previous studies (25). These findings suggest that, beyond adapting to abnormal Achilles tendon loading, individuals post-stroke may have an increased susceptibility to Achilles tendon pathology, potentially due to the influence of metabolic syndrome. The presence of tendinopathy in our participants with post-stroke hemiparesis remained uncertain. Although none of our participants reported pain in either leg, attributing any potential pain specifically to tendinopathy would be challenging in persons post-stroke. This ambiguity stems from the likelihood of potential impaired pain perception related to stroke lesion (45, 46), which could confound the identification of tendinopathy as the underlying cause of pain.

Our research has potential to impact rehabilitation strategies for individuals with post-stroke hemiparesis. By identifying tendon morphological changes as potential contributors to altered walking mechanics, our findings advocate for a more comprehensive treatment approach that includes both muscle and tendon function, especially in addressing insufficient paretic propulsion during gait. In addition, given our focus on higher-functioning stroke participants—demonstrated by the ability to complete a 10-Meter Walk Test, FMA-LE scores (22.6 out of 34), lower MAS (0.8 out of 4), and community ambulation speed (47)—we recognize that individuals with lower functional levels may exhibit more pronounced or different Achilles tendon changes. In reviewing the limited literature, previous studies examining Achilles tendon morphology and mechanical properties did not offer specific details about the functional levels of their post-stroke group beyond their ability to walk independently or with a cane (12, 13, 16). Other functional measures, such as FMA, overground walking speed (as used in our study), balance, or the ability to perform activities of daily living, were not reported. It is important to note that the functional levels of stroke survivors can vary widely depending on the severity and location of the stroke, as well as individual factors such as age, pre-stroke health status, and access to rehabilitation services. The lack of detailed information about the functional levels of the participants in these studies limits the ability to make comparisons to other stroke populations or to draw conclusions about the relationship between functional level and Achilles tendon morphology or mechanical properties. Expanding research to include participants across a broader range of functional abilities and assessing both muscle co-activation patterns during gait and tendon integrity (e.g., mechanical properties and morphology) could help establish clinically meaningful thresholds or minimal important differences in muscle-tendon function post-stroke. Tracking these adaptations over time, particularly in the acute or subacute phases of stroke recovery, would enhance understanding of rehabilitation needs at various recovery stages.

A limitation of our study is the higher body weight observed in the stroke group, although the difference between groups was not statistically significant. Higher body weight is a common outcome post-stroke due to pre-existing obesity and/or reduced physical activity. Increased body weight may elevate Achilles tendon loading, while decreased activity levels could lower it. Additionally, as our previous work showed pronounced tendon thickening at the insertion site (17), this study focused on the distal portion of the Achilles tendon; therefore, examining the entire tendon, including the proximal region, is warranted. Moreover, while stroke participants exhibited lower PSFR and increased tendon thickness in the paretic limb, the specific thresholds for defining abnormal morphology remain unclear. Although tendon mechanical properties have been previously studied in individuals post-stroke, this study did not evaluate them in conjunction with Achilles tendon morphology. Key biomechanical factors during walking—such as propulsive forces, joint kinematics and kinetics, and muscle co-activation—were also not assessed. To address these gaps, future research should prioritize longitudinal analyses to monitor tendon morphology changes across multiple sites, establish diagnostic thresholds for PSFR and tendon thickness, and consider the impact of factors such as metabolic syndrome in individuals post-stroke. Controlling for body weight and activity levels between stroke and non-stroke participants will also be critical. Building on our findings, it is essential to adopt a more comprehensive research design that integrates tendon morphology, tendon mechanical properties, and biomechanical factors during walking—including kinetics, kinematics, and muscle activation patterns. This holistic approach will provide deeper insights into the mechanisms underlying tendon health and gait performance in individuals post-stroke.

## 5 Conclusions

Individuals with post-stroke hemiparesis exhibited increased thickness both 2 cm above the insertion and at the insertion site of the Achilles tendon within the paretic limb, along with marked disorganization of collagen fiber bundles at the insertion point. These morphological changes are likely the result of a complex, abnormal loading environment, influenced by factors such as increased plantarflexor muscle tone, muscle co-activation, and muscle atrophy and disuse in the paretic limb. Such changes in tendon structure may lead to increased compliance of the Achilles tendon during gait, impairing force transmission and propulsion in the paretic limb, and ultimately contributing to slower walking speed post-stroke. These findings highlight the importance of a comprehensive treatment approach focused on improving neuromuscular control in this population. To enhance walking speed in individuals post-stroke, it is important to assess and address Achilles tendon integrity, particularly in those with increased plantarflexor muscle tone, inappropriate muscle co-activation patterns, insufficient paretic propulsive forces, and/or metabolic syndrome. Targeting these interconnected impairments may facilitate better recovery of functional gait and overall mobility in this population. The clinical implications of our research findings warrant further investigation to optimize rehabilitation strategies.

## Data Availability

The raw data supporting the conclusions of this article will be made available by the authors, without undue reservation.
